# C-Met as a Key Factor Responsible for Sustaining Undifferentiated Phenotype and Therapy Resistance in Renal Carcinomas

**DOI:** 10.3390/cells8030272

**Published:** 2019-03-22

**Authors:** Paulina Marona, Judyta Górka, Jerzy Kotlinowski, Marcin Majka, Jolanta Jura, Katarzyna Miekus

**Affiliations:** 1 Department of General Biochemistry, Faculty of Biochemistry, Biopphisics and Biotechnology, Jagiellonian University, Gronostajowa Street 7, 30-387 Krakow, Poland; paulina.marona@doctoral.uj.edu.pl (P.M.); judyta.ciesla.gorka@gmail.com (J.G.); j.kotlinowski@uj.edu.pl (J.K.); jolanta.jura@uj.edu.pl (J.J.); 2 Department of Transplantation, Jagiellonian University Medical College, Jagiellonian University, Wielicka 265, 30-663 Krakow, Poland; mmajka@cm-uj.krakow.pl

**Keywords:** C-Met receptor, therapy resistance, epithelial to mesenchymal phenotype, EMT, differentiation, renal carcinoma, RCC.

## Abstract

C-Met tyrosine kinase receptor plays an important role under normal and pathological conditions. In tumor cells’ overexpression or incorrect activation of c-Met, this leads to stimulation of proliferation, survival and increase of motile activity. This receptor is also described as a marker of cancer initiating cells. The latest research shows that the c-Met receptor has an influence on the development of resistance to targeted cancer treatment. High c-Met expression and activation in renal cell carcinomas is associated with the progression of the disease and poor survival of patients. C-Met receptor has become a therapeutic target in kidney cancer. However, the therapies used so far using c-Met tyrosine kinase inhibitors demonstrate resistance to treatment. On the other hand, the c-Met pathway may act as an alternative target pathway in tumors that are resistant to other therapies. Combination treatment together with c-Met inhibitor reduces tumor growth, vascularization and pro-metastatic behavior and results in suppressed mesenchymal phenotype and vascular endothelial growth factor (VEGF) secretion. Recently, it has been shown that the acquirement of mesenchymal phenotype or lack of cell differentiation might be related to the presence of the c-Met receptor and is consequently responsible for therapy resistance. This review presents the results from recent studies identifying c-Met as an important factor in renal carcinomas being responsible for tumor growth, progression and metastasis, indicating the role of c-Met in resistance to antitumor therapy and demonstrating the pivotal role of c-Met in supporting mesenchymal cell phenotype.

## 1. Introduction

The formation of a malignant tumor is a complex and multi-stage process. This process is dependent on tumor cells gaining various qualities that enable them to leave the original tumor and subsequently inhabit the place where cancer metastasis has developed. The regulation of this process takes place in many stages and depends among others on the presence and activity of the receptors on the surface of cells. Receptors have particular importance in the process of growth, invasion and metastasis possess tyrosine kinase activity [1]. One of the well-known receptors connected with tumor growth and the metastatic process is the c-Met receptor, a product of the proto-oncogene *MET.* The activation of the c-Met receptor through its ligand, hepatocyte growth factor (HGF), also known as the scatter factor (SF), leads to the stimulation of various biological effects. Under normal conditions, this receptor takes part in embryogenesis, development of organs, differentiation of i.a. muscular and nerve cells, as well as regeneration of the liver [2,3,4]. In tumor cells’ overexpression or incorrect activation, this leads to the stimulation of proliferation, survival and an increase of motile activity. This receptor is also described as a marker of cancer initiating cells. The latest research shows that the c-Met receptor has its influence on the development of resistance to targeted cancer treatment [4,5].

In this review, we present recent advances that have been made in the study of the c-Met receptor in kidney tumors, review the mechanisms underlying therapy resistance and summarize the evidence on the role of the c-Met receptor in sustaining the undifferentiated mesenchymal phenotype of cancer cells.

## 2. C-Met Receptor

C-Met is expressed by epithelial cells of many organs, including the liver, pancreas, prostate, kidneys, lungs and bronchus. It is localized on the cells’ membrane and is activated upon binding of Hepatocyte Growth Factor (HGF) or its splicing isoforms—the only known endogenous ligands so far [6]. C-Met activation by HGF induces its tyrosine kinase catalytic activity which triggers transphosphorylation of the tyrosine Tyr 1234 and Tyr 1235, initiating a whole spectrum of biological activities including regulation of proliferation, cell motility or cell cycle progression [7]. 

Such a broad spectrum of HGF/c-Met actions led to the investigation of both *MET* gene expression and c-Met activity in tumor cells. In fact, c-Met is deregulated in many types of human malignancies, kidney, liver, stomach, breast and brain cancers. What is more, abnormal c-Met activation in cancer specimens correlates with poor prognosis, where active receptor triggers tumor growth, angiogenesis and metastasis. Today, *MET* is considered as a proto-oncogene and its mutations or overexpression leads to aberrant, often constitutive activation of the HGF/c-Met axis [8,9]. Autocrine or paracrine activation of c-Met is directly related to the promotion and progression of tumors in organs such as: liver, lung, colon, breast, pancreas, ovary, prostate, stomach and kidney [6,10,11,12].

## 3. C-Met Receptor and Kidney Tumors

In the adult human kidney, the c-Met receptor is expressed in tubular epithelial cells where it stimulates the growth of renal tubular cells [13,14,15,16]. Proper c-Met function is also important for the induction of branching tubulogenesis during tubule repair following ischemic and chemical injuries or contralateral nephrectomy [17,18,19]. 

Renal cell carcinomas (RCC) are divided into several major subtypes: the most common is clear cell RCC (ccRCC, 75% of cases), papillary RCC (pRCC 15%) and chromophobe RCC (5%) [20]. Their common feature is a well-developed vascularization and, interestingly, upregulation of the c-Met receptor level compared to the healthy kidney [21,22]. 

It has been shown that c-Met is overexpressed in renal cell carcinomas and its phosphorylation is associated with progression of the disease [23,24].

ccRCC creates extremely vascularized tumors due to frequent loss of function mutation in the von Hippel-Lindau tumor suppressor gene (VHL) located on chromosome 3p which is responsible for regulating the stability of hypoxia-inducible factor 1 (HIF-1) [25]. The loss of VHL activity results in HIFs accumulation which leads to excessive secretion of vascular endothelial growth factor (VEGF) or platelet-derived growth factor (PDGF), as well as receptors that are potentially important in ccRCC oncogenesis [26], resulting in increased ability of tumor cells to metastasis. It is also known that HGF via its receptor c-Met may regulate VEGF expression and promote angiogenesis [27]. Furthermore, it was shown that VHL mutation together with hypoxia lead to increased HGF and c-Met expression in ccRCC [28,29]. In addition, HIF-1 during hypoxia regulates the expression of 1–5% of human genes, inter alia c-Met and VEGF [30]. Thus, tumor angiogenesis together with c-Met receptor have become a major focus of targeted therapy for RCC.

In 1996, Natali and coworkers presented for the first time a comparison of c-Met expression between the normal human kidney, urogenital tissue and 50 different histotypes of kidney tumors [23] (Table 1). They showed a low level of c-Met receptor in tissues of the genitourinary tract in contrast to a relatively high expression in tumor tissue. Such observations allowed scientists to link HGF/c-Met axis with induction and progression of renal cancer cells [23]. Results published in the following year by Pisters et al. also confirmed this observation. Out of 41 samples from normal and cancerous human tissue analyzed by immunohistochemical (IHC) staining, 28 were positive for c-Met. Authors detected c-Met in various subtypes of kidney tumors and correlated its amount with renal cell carcinoma (RCC) stage assessed by the Fuhrman grading scale [31]. Nuclear grading of RCC, the so called Fuhrman scale, was initially described in 1982 after prognostic evaluation of morphologic parameters in 103 patients diagnosed with renal cell carcinoma [32]. Four nuclear grades (1–4) were defined in order of increasing nuclear size, irregularity and nucleolar prominence. Importantly, nuclear grade was more effective than each of the other parameters (e.g. tumor size, cell arrangement or cell type) in predicting the development of distant metastasis following nephrectomy. For example, among 45 tumors classified as nuclear grade 1, none of them metastasized for at least five years, whereas 50% of the higher-grade tumors did [32]. As demonstrated by Pisters and coworkers, according to the Fuhrman nuclear grade, c-Met was detected in 20 cases out of 24 (83%) in 3 and 4 grades, while only in 8 out of 17 (47%) cases of grades 1 and 2. Thus, the biological significance of the HGF/ c-Met pathway in the development, progression and metastasis of kidney cancer was confirmed by the demonstration of the correlation between c-Met expression and tumor progression [31]. Subsequent studies have also described a high amount of c-Met receptor in renal tumors. In 2006, Choi and coworkers detected by IHC staining exaggerated c-Met expression in 90% of papillary RCC, 100% of collecting duct carcinoma and 92% of urothelial carcinoma [33]. *MET* expression was more heterogenous in clear cell RCC and chromophobe RCC, where c-Met protein was detected by IHC staining in 45% and 8% of samples, respectively. Next, Choi and coworkers correlated the expression of c-Met with the Fuhrman grade in RCC specimens, together with clinical and pathological parameters related to the invasiveness or aggressiveness of tumors. Importantly, they demonstrated that c-Met expression in clear cell RCC correlated with high nuclear grade: stages I/II = 18%/29% versus stages III/IV = 58%/94% (*p* < 0.001). Additionally, the level of c-Met protein correlated with tumor necrosis (*p* < 0.001) and the presence of lymphatic invasion (*p* = 0.016) [33]. A recent study done by Gibney et al. also confirmed this data. Among 303 RCC tumors paired with adjacent normal renal tissue, higher c-Met expression was detected in all RCC subtypes than in the adjacent normal renal tissue (*p* < 0.0001). Additionally, c-Met levels correlated with higher Fuhrman grades and more advanced disease stages [21]. However, the differences were not significant; higher level of c-Met in grades III/IV = 39 versus grades I/II = 33 and III/IV = 38 versus stages I/II 34, which implicates that c-Met might be important in the early stages of tumorigenesis. 

### 3.1. C-Met is Associated with an Advanced Stage and Poor Survival of Patients

Although Fuhrman nuclear grading correlates with renal carcinoma severity, the patients’ survival rate should be considered as the most important parameter. As such, the expression of c-Met should also be correlated with survival analysis. Patients’ overall survival is affected by many factors, like: the degree of local tumor advancement (T category), the presence of metastases in the lymph nodes (N category) or the existence of distant metastases at the time of cancer diagnosis (M category) [34]. The staging system analyzing all the above mentioned criteria—a Classification of Malignant Tumors (TNM) system —was developed by the American Joint Committee on Cancer (AJCC). The TNM classification was developed as a tool for physicians to stage different types of tumors based on certain standardized criteria and is now the most commonly applied staging system used by medical professionals and scientists around the world. Analyses carried out by Pisters et al. in 1997 revealed not only a correlation between c-Met expression and high nuclear grade (*p* = 0.008), however also with TNM stage (*p* = 0.001) and disease-specific survival or metastases [31]. A later study of 303 RCC samples demonstrated that higher c-Met expression correlated with worse disease-specific survival (*p* = 0.0091) and was an independent predictor of survival [21]. More recently, analysis of 572 clear-cell RCC tumors demonstrated that only 17% were negative for c-Met expression, whereas 32% showed high protein levels. High c-Met was associated with an aggressive phenotype and an unfavorable patient outcome [35] (Table 1). In accordance with these results, better overall survival was demonstrated for patients characterized by RCC tumors with low c-Met expression in comparison to high c-Met expressing subjects [36]. Interestingly, in 58% of cases (18 out of 31 cases), high c-Met expression in primary tumors correlated with its amount in metastases. However, there was no such relationship in 42% of specimens (13 out of 31) [37]. Conflicting results were also published by Miyata and coworkers. In the latter analysis, total c-Met expression was not associated with a clinical and pathological characteristic, however, c-Met was associated with an advanced stage and the presence of metastases [24]. 

### 3.2. Germline and Somatic Mutations of C-Met Receptor in Renal Carcinomas

Most of the renal tumors revealed are sporadic. However, 4% of all detected kidney cancers can be family-related among others in the group of von Hippel-Lindau or the Brit-Hogg-Dube band [28,38]. C-Met is a protein that in humans is encoded by the *MET* gene and is located in the 7q31 locus of chromosome 7 [39]. Patients with tumors are identified by proto-oncogene mutations. Mutations that cause constitutive phosphorylation of the c-Met receptor are identified in papillary kidney cancer at 13% (17/129) [40]. Hereditary papillary renal carcinoma (HPRC) is inherited and autosomal dominant transmission occurs with reduced penetrance. In the case of malignant tumors, chromosomal trisomy 7, 16 and 17 occurs and, among men, the loss of the Y chromosome. Missense mutation is a point mutation in which a single nucleotide change results in a codon that codes for a different amino acid and, when located in the tyrosine kinase domain of the *MET* gene, it leads to constitutive activation of the c-Met protein and papillary renal carcinomas [41]. Missense mutations involve replacing histidine 1112 with leucine (H1112L), histidine 1124 with aspartic acid (H1124D) or tyrosine 1248 with aspartic acid (Y1248D). The mutations in the tyrosine kinase domain (amino acids 1110 to 1268) lead to the inheritance of papillary renal carcinoma [40]. In the case of some patients with this type of tumor, there is a chromosomal aberration involving chromosomal translocation which leads to fusion with the *MET* gene and *BAIAP2L1* or *C8orf34*. This results in protein coding with motifs that facilitate protein dimerization, fusion and ultimately promote c-Met receptor kinase activity, even in the absence of the HGF ligand [42]. The promotion of c-Met kinase activity in papillary renal carcinoma also occurs during the V1110I mutation where the change of valine to leucine in the conserved ATP binding pocket of the c-Met receptor enhances its kinase activity [43]. With the increase in c-Met protein levels, there is also an increase in the number of *MET* copies in the primary and metastatic tumors. According to Macher-Goeppinger et al., an increase in *MET* copy numbers was detected in 310 cases out of 572. The number of copies per nucleus ranged from less than 2 (262/572 cases), from 2 to 4 (272/572) and the remaining 7% (38/572) more than 4 copies. *MET* copy number correlates with dedifferentiation (*p* < 0.001), higher tumor extent (*p* < 0.001), positive lymph node status (*p* = 0.006) and distant metastasis (*p* = 0.02). There was no consistent relationship between the number of *MET* copies and the age of the patient [35] (Table 1).

Further studies showed that the increase in *MET* copy numbers correlates with poorer survival and distant metastases in clear cell renal cell carcinoma [21]. 

### 3.3. Phosphorylation of C-Met in Renal Carcinomas

All the above mentioned results analyzed the amount of c-Met protein, which may not be sufficient for a complete understanding of RCC biology. Today, we know that HGF binding by c-Met leads to receptor homodimerization and autophosphorylation, which is a key event in its activation. The study performed by Miyata et al. showed for the first time the expression of active c-Met receptor - phosphorylated at Tyr 1349 residue. This active pY1349 c-Met receptor was mainly detected in cancer cell membranes and was positively associated with grade (*p* = 0.004), lymph node metastases (*p* = 0.014), distant metastases (*p* = 0.021) and pT-stage (*p* < 0.001). Such tumors were also characterized by higher proliferation rate (*p* < 0.001) and bigger tumor diameter (*p* = 0.017) in comparison to samples without phosphorylated c-Met [24]. Additionally, positive expression of pY1349 c-Met was a significant and an independent predictor of cause-specific survival (*p* = 0.028) [24] (Table 1). 

Important data concerning c-Met activation in RCC comes from in vitro studies done by Nakaigawa et al. [28]. They reported that c-Met in ccRCC cell line was constitutively activated without HGF stimulation due to the inactivation of the *VHL* proto-oncogene. In this model, c-Met was constitutively active even at an early stage of tumor formation, however constitutive phosphorylation of the c-Met protein in vitro was inhibited by wild-type *VHL* gene delivery. Additionally, c-Met protein activated by the inactivation of the *VHL* gene modified cell adherence, including N-cadherin and beta-catenin. Scientists concluded that inactivation of the *VHL* gene induced constitutive phosphorylation of the c-Met protein and modified intercellular adherence structure, triggering cell growth released from contact inhibition, finally resulting in tumorigenesis [28]. 

The study performed by our group also showed that c-Met receptor phosphorylation is positively associated with tumor growth and its vascularization, lung metastasis and ccRCC cells’ migratory potential [44].

## 4. Resistance to Anti-Cancer Therapies in RCC

The c-Met receptor has become a therapeutic target in many cancers as well as in kidney cancer. Selective inhibitors of c-Met are currently in the clinical development phase. Inhibiting abnormal c-Met activity can significantly increase patient survival, reduce the rate and extent of metastasis and prolong the lives of patients who are often diagnosed only at advanced stages of cancer. Unfortunately, regardless of this, the therapies used so far using c-Met tyrosine kinase inhibitors demonstrate resistance to treatment [48].

Resistance to anti-cancer therapies is a known phenomenon that is still not fully understood. Many tumors, despite the first good response, over time develop strong resistance to the drugs used [49,50,51]. Mechanisms underlying resistance are based, among other things, on drug inactivation, alternative signaling pathways activation, drug target alteration and the epithelial-mesenchymal transition [52,53]. 

RCCs are usually not sensitive to chemotherapy and the majority of drugs used in RCC target angiogenic pathways. Up to now, several drugs have been created that target the VEGF and PDGF pathways. Bevacizumab and nivolumab are monoclonal antibodies targeted at VEGF and PD-1. Everolimus and temsirolimus, second line mTOR inhibitors, downregulate both cell proliferation and angiogenesis. Whereas, drugs such as sunitinib, sorafenib, pazopanib, axitinib, lenvatinib and cabozantinib are the largest family of tyrosine kinase inhibitors (TKIs) [54,55,56,57,58,59,60]. TKIs act as anti-angiogenic agents through the inhibition of tumor endothelium growth and cell survival signaling impairment [56,57]. As multi-targeted agents, they inhibit number of receptors with varying potency such as VEGFRs, PDGFR and, recently, also c-Met, which are involved in several signaling pathways [61].

Despite the overall longer survival rate and first good response, eventually all patients with metastatic RCC show resistance to the anti-angiogenic therapy, especially to TKIs. Suppressing the VEGF pathway initially decreases tumor progression and vascularization, however after a couple of months of treatment, rapid recurrence occurs [49,62]. Prolonged exposure of tumor cells to sunitinib results in quick drug resistance development. Interestingly, in vitro studies have shown that such resistance may be transient and cells sensitivity is restored after some period of drug-free culture, both in selected RCC patients and tumor models, suggesting epigenetic tumor adaptation to TKIs [63]. 

**Figure 1:**
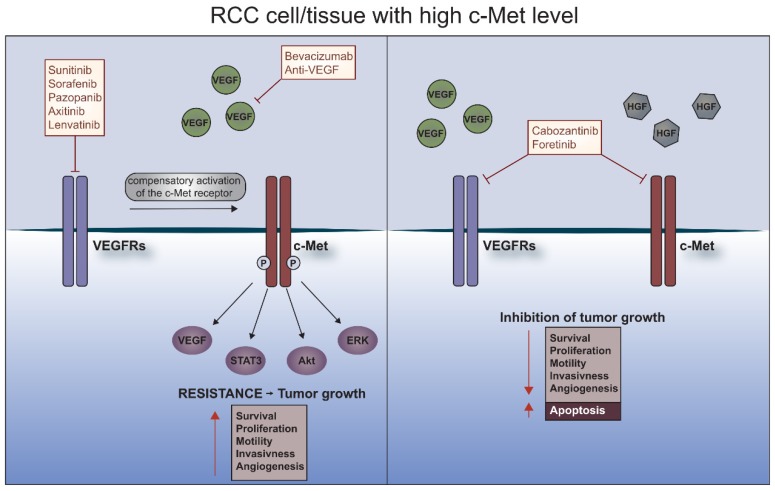
C-Met receptor and resistance to anti-cancer therapies in RCC. RCC tissue is characterized by a high level of c-Met receptor. Under treatment with VEGFRs inhibitors such as sunitinib, sorafenib, pazopanib, axitinib or lenvatinib, c-Met is phosphorylated and activates many downstream pathways involved in survival, proliferation, motility, invasiveness and angiogenesis which leads to the development of resistance. Dual blockade of the c-Met receptor and VEGFRs causes induction of apoptosis and inhibition of tumor growth.

The main mechanisms underlying the emergence of acquired resistance to the used anti-angiogenic therapies is the epithelial to mesenchymal transition [53] and the compensation of blocked receptors as well as the activation of alternative proteins or signaling pathways such as the HGF/c-Met pathway, which could drive tumor angiogenesis or growth independently from VEGFRs [64,65,66]. Also, lack of influx of endothelial cells caused by lack or blockade of VEGF may cause vasculogenic mimicry within tumors that takes the role of endothelium and forms structures similar to blood vessels [67,68] (Figure 1).

### 4.1. C-Met Receptor and Therapy Resistance

Shojaei et al. were first to show that the HGF/c-Met pathway acts as an alternative pathway in sunitinib resistant tumors [64]. They demonstrated that not only tumor cells, however also stroma (non-tumor compartment) contribute to the development of resistance to sunitinib therapy due to upregulated HGF expression in stroma cells of mesenchymal origin. The main target for secreted HGF were c-Met on the surface of endothelial cells within the tumor, which indicates that the tumor vasculature mainly responds with the resistance to the sunitinib therapy by a shift to the alternative signaling pathway [64]. It is worth noting that studies on IL8 role in resistance to sunitinib have shown that endothelial cells are sensitive to sunitinib whereas tumor cells are only minimally [69]. Interestingly, after the combination of sunitinib and a highly selective c-Met inhibitor, PF-04217903 growth inhibition was observed, however, only in sunitinib resistant tumors [64]. Similar results were observed in a pancreatic tumor study [65]. It was found that after sunitinib treatment of transgenic RIP-Tag2 mice, tumor growth slowed and reduced vascularity, however there was an increased hypoxia together with HIF1α accumulation, upregulation of c-Met receptor and epithelial to mesenchymal transition (EMT) markers such as Snail1, N-cadherin and vimentin. However, after the introduction of c-Met inhibitor PF-04217903 to crizotinib or sunitinib treatment, invasion and liver metastasis as well as c-Met and EMT markers expression were reduced [65]. The inhibition of both signaling pathways by cabozantinib (XL184), a dual c-Met and VEGFRs inhibitor, also reduced tumor growth, invasion, metastasis and prolonged survival of RIP-Tag2 mice [65]. Studies conducted using a 786-O cell line characterized by a high level of c-Met showed that the combination of anti-angiogenic agent axitinib (which targets all VEGFRs) with crizotinib inhibits tumor growth and reduces vascularization compared to individual therapy with these drugs. The same effect was observed in the sunitinib resistant RP-R-01 ccRCC PDX model, however not in the sunitinib sensitive one (with low c-Met levels) [70]. Similar results, showed by Zhou et al., indicate that resistance to sunitinib was induced by upregulation of c-Met and AXL receptors which promotes pro-metastatic behavior and angiogenesis in ccRCC [66]. Furthermore, chronic exposure to sunitinib promotes EMT signaling, upregulates levels of c-Met downstream proteins such as ERK, Akt and Src and elevates cell migration and invasion in vitro and in vivo [66]. EMT signaling was impaired after AXL or c-Met suppression with specific shRNA, which indicates that chronic sunitinib treatment is AXL and c-Met dependent. Also, tumor angiogenesis (tube formation) was reduced after AXL or c-Met inhibition in RCC cell line 786-O co-cultured with endothelial cells HUVEC [66]. Moreover, administration of cabozantinib results in suppressed EMT phenotype and VEGF *secretion* and rescues sunitinib resistance. Treatment with cabozantinib reduced tumor size during in vivo experiments [66]. Interestingly, it was shown that HIF1α directly activates the expression of AXL by binding the hypoxia-response element to the AXL promoter. AXL together with its ligand growth arrest-specific 6 (GAS6) form a complex with SRC proto-oncogene and activate c-Met in an HGF-independent manner which leads to increased migration and invasion of ccRCC [71]. After using two inhibitors specific for AXL (sAXL) and c-Met (ARQ197), it was shown that sAXL therapy reduced invasion by 70% while c-Met inhibition only by 40%. This suggests that GAS6/AXL signaling activates c-Met to maximize invasion through noncanonical signaling mechanisms in RCC [71]. Recently, c-Met was proposed as a prognostic marker in patients with metastatic RCC (mRCC) treated with sunitinib due to the correlation of higher c-Met expression with faster resistance to sunitinib [72].

The undeniable benefits of combining anti-angiogenic therapies with blocking the c-Met receptor have prompted scientists to create one drug that is a combination of VEGFR and c-Met inhibitors. Cabozantinib is a novel TKI that targets VEGFR-2, c-Met, AXL, RET, KIT, Tie-2 and FLT3, which are involved in invasiveness, metastasis and survival [73]. It was approved in 2016 by the US Food and Drug Administration (FDA) for treatment of patients with mRCC following anti-angiogenic therapy [74]. In phase I of the clinical trial on 25 patients pretreated with VEGF or mTOR inhibitors, after cabozantinib administration (maximum 140 mg daily), a 28% response rate with a median progression-free survival (PFS) of 12.9 months was observed [75]. The phase II randomized trial in metastatic RCC cabozantinib (79 patients) versus sunitinib (78 patients) revealed significantly longer median cabozantinib PFS of 8.6 months compared with 5.3 months with sunitinib and overall survival (OS) was 26.6 versus 21.2 months for cabozantinib [76]. Finally, a phase III randomized trial METEOR investigated cabozantinib versus everolimus in 658 patients with PFS 7.4 versus 3.9 and OS 21.4 versus 16.5 for cabozantinib [77]. Cabozantinib seems to be a very good second-line therapy for patients with mRCC, having very good results of PSF or OS in comparison with other therapeutics used in the treatment of RCC due to wide spectrum of targeted kinases, especially c-Met and AXL. 

Lastly, foretinib, a multikinase inhibitor targeting c-Met, VEGF, AXL, RON and Tie-2, was tested on patients with papillary renal cell carcinoma. pRCC is often characterized by mutations or amplification in the *MET* gene. In the phase II randomized clinical trial on foretinib, patients were classified based on c-Met pathway activation: germline or somatic mutation, *MET* [7q31] amplification or gain of chromosome 7. Foretinib demonstrated activity in patients with pRCC with median PFS 9.3 and a high response rate in patients with germline *MET* mutations [78]. Despite a relatively good response to the drug and several clinical trials, work on foritinib has been discontinued.

Anti-angiogenic therapies of RCC, based on TKIs, such as sunitinib or axitinib show significant benefits in vasculature reduction and decrease of tumor size, especially in ccRCC patients. However, despite the initial good response, relapse occurs due to the acquisition of resistance, resulting in a shift to alternative pathways that will support and promote angiogenesis. The c-Met/HGF signaling pathway plays a significant role in tumor and endothelial cells where it acts as a potent pro-angiogenic trigger. New TKIs, which target not only VEGFR, however also the c-Met receptor, appear to be a response to the problem of resistance, showing a similar tolerance and toxicity as first-line TKIs, yet a superior PFS response and efficiency (Figure 1).

### 4.2. Epithelial to Mesenchymal Transition in the Acquisition of Therapeutic Resistance

Developing resistance to treatment that limits the success of cancer therapy is one of the most difficult problems of the contemporary oncology. Despite significant progress in the use of chemotherapy over the past 40 years, most chemotherapeutic regimens have failed to provide satisfactory therapeutic outcomes due to relapse caused by resistance to treatment. Understanding the molecular mechanisms that cause resistance to a therapy is of great importance for the development of alternative therapies and the improvement of clinical benefits. Most of the mechanisms of resistance include, among others, secondary mutations of inhibited pathways or amplifications of growth factor receptors. However, it seems that the resistance acquisition process is more complex and dependent on a number of changes in the tumor cell itself and in the tumor microenvironment.

The acquisition of therapeutic resistance and progression to distant metastatic disease might be a result of the epithelial to mesenchymal transition (EMT) process.

The epithelial to mesenchymal transition was observed for the first time in the context of embryonic development, where it regulates key morphogenetic steps such as gastrulation and neural crest formation [79]. EMT is also a key element of tumor progression in which epithelial cells acquire mesenchymal features facilitating their migration, invasion of neighboring tissues and metastasis. During EMT, clearly polarized epithelial cells with high expression of E-cadherin and other proteins characteristic for epithelial cells phenotype, under the influence of growth factors, cytokines and other environmental factors change their morphology to an elongated one and become migrating cells with the expression of i.e. vimentin, fibronectin, N-cadherin and a decrease of E-cadherin [80]. E-cadherin is a transmembrane protein located in the adherens junctions, playing an important role in the sustaining of the epithelial morphology and the inhibition of the invasion and metastasis of the cancer cells. Loss of the expression of E-cadherin is a well-known sign of the EMT, which may cause the transformation of the non-malignant tumor into a locally aggressive and invasive form [81]. It has also been demonstrated that the induction of EMT in epithelial tumor cells results in the acquisition of features of tumor-initiating cells, also designated as cancer stem cells (CSCs) [82,83]. Cancer cells with stem cell characteristics have been found to be enriched in the residual tumors remaining after standard chemotherapeutic treatments [84]. 

## 5. C-Met Receptor as a Marker of Undifferentiated Cells

The c-Met receptor has been postulated as an essential factor responsible for the functional cancer stem cell (CSC) phenotype in some tumors and as a CSC factor, it is believed to be responsible for therapy resistance [85]. 

The results obtained by our team and other research groups have shown that the c-Met receptor is also important in the process of cell differentiation and resistance to chemotherapy for some tumors. 

In the biology of rhabdomyosarcoma, one of the most common tumors found in the soft tissues of children, the c-Met receptor is very important for the process of proliferation, survival and resistance to chemotherapy [86,87,88,89]. RMS cells lacking the c-Met receptor were more differentiated and had a significantly reduced level of MyoD transcription factor, the marker for undifferentiated cells [86]. It has also been shown that in the RMS cells subjected to a differentiation process, they displayed a significant decrease of MyoD and myogenin factors that are characteristic of the rhabdomyosarcoma. The process of differentiation decreases the c-Met receptor level significantly. What is more, these cells changed their morphology to a more elongated one and lost their ability for active migration. The obtained results clearly indicated the significant role that the c-Met plays in the sustaining of the RMS phenotype. The research has proven that the c-Met receptor is a significant factor for sustaining the rhabdomyosarcoma cells in an undifferentiated state. RMS may derive from a differentiation defect of either mesenchymal stem cells (MSC) or myogenic progenitors [90]. It has been shown that activation of c-Met signaling blocks myogenic differentiation of RMS and may be responsible for the impairment of myogenic differentiation of MSC and, as a consequence, may lead to oncogenic transformation towards embryonal RMS development [88]. It has been shown that in muscle satellite cells, c-Met activation inhibits the exit from the cell cycle and delays myogenic differentiation [91].

The silencing of the c-Met receptor results in changes of the phenotype in other types of tumor cells. Similar research has shown that the silencing of the c-Met receptor in cervical cancer cells with epithelial origin induced the increase in the level of E-cadherin [92]. Decreased c-Met level also increases the level of E-cadherin in tumors of NOD-SCID mice. The cells with silenced expression of the c-Met receptor have a decreased level of Slug transcription factor, one of the repressors of E-cadherin expression [92,93].

**Figure 2:**
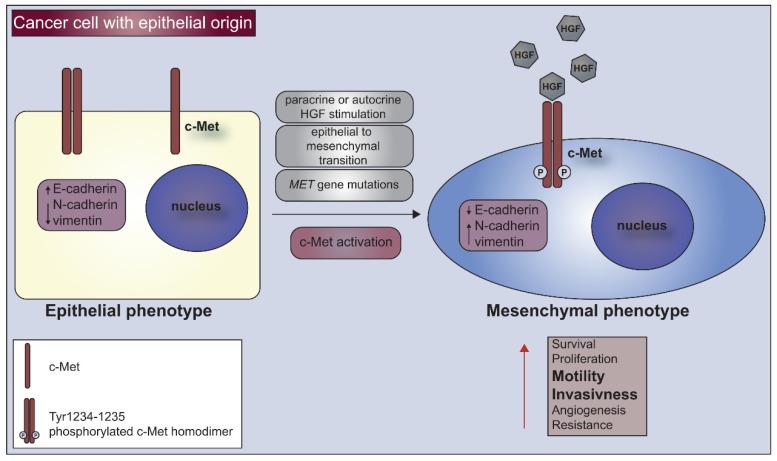
C-Met receptor as a marker of undifferentiated cells. Cancer cells with epithelial phenotype under hepatocyte growth factor (HGF) stimulation, epithelial to mesenchymal transition (EMT) or *MET* gene mutations acquire characteristics of undifferentiated, mesenchymal cells. Activation of c-Met leads to downregulation of E-cadherin and upregulation of N-cadherin and vimentin which cause increased survival, motility and invasiveness of cancer cells.

Interesting results also came from research on lung cancer where the reinstitution of the E-cadherin expression increased the cancer cells’ sensitivity to the cytostatic drug, gefitinib [94]. Summarizing the above, our research has shown that the c-Met receptor is responsible for sustaining the mesenchymal phenotype in cervical carcinoma cells (Figure 2).

However, the role of the c-Met receptor in the acquisition of mesenchymal phenotype is not well understood for kidney cancer. Our group has already shown that in an in vitro model of clear cell renal cell carcinoma cells and in the normal human renal cell line HEK293, increased level and phosphorylation of c-Met correlates with the acquisition of mesenchymal cell characteristics, i.e. an increase in the migration activity, the suppression of E-cadherin and upregulated β-catenin and vimentin [44]. The increased c-Met activation was the result of the lack of anti-inflammatory MCPIP1 protein that controls the inflammatory process. Our observation showed that low level of MCPIP1 in ccRCC led to an increased tumor growth, vascularization and lung metastasis in both NOD-SCID and Nude mice together with the loss of epithelial phenotype of cancer cells [44]. 

The presence of the c-Met receptor is crucial not only for the high level of proliferation and initialization of tumor growth in in vivo conditions, however is also a significant factor in sustaining the undifferentiated/mesenchymal phenotype of cancer cells (Figure 2).

## 6. Conclusions

The c-Met receptor plays a significant role in healthy kidney and RCC development. Paracrine or autocrine HGF stimulation, EMT or *MET* gene mutations lead to c-Met activation and stimulation of various pathological effects, such as proliferation, survival, angiogenesis and increased motile activity. In addition, this receptor is described as a marker of cancer initiating cells. All this leads to tumor growth and progression.

Here we showed that c-Met level is upregulated in RCC tumor tissue compared to normal kidney and increases during RCC progression. Frequent VHL mutation and hypoxia lead to increased c-Met expression in clear cell RCC. In papillary RCC, missense mutation of the *MET* gene causes constitutive activation of the c-Met protein, which correlates with poor survival rates. The c-Met receptor becomes one of the main factors involved in acquiring resistance to targeted therapies used in RCC. Mechanisms underlying therapy resistance consist of the compensation of blocking VEGFRs by activating other receptors involved in the regulation of angiogenesis such as the c-Met. Therefore, combination treatment of anti-angiogenic drugs with c-Met inhibitor reduces tumor growth, vascularization and VEGF secretion. Moreover, the role of the c-Met in tumor progression is extremely significant through its impact on sustaining the undifferentiated mesenchymal phenotype of cancer cells, which consequently is responsible for therapy resistance. 

Taken together, c-Met is an important factor involved in the acquisition of resistance to targeted therapies, sustaining mesenchymal, undifferentiated phenotype, and increased renal carcinomas’ growth, progression and invasiveness. 

## Figures and Tables

**Table 1:** C-Met expression and phosphorylation in renal cell carcinomas (RCC) subtypes. The correlation between c-Met expression, protein level, receptor activation and different stages of the tumor progression in patients with RCC.

Study (Year)	Type	c-Met Detection Method	Expression c-Met (Number Positive/Number Tested)	c-Met Expression in Percent	Major Conclusion
Natali et al. (1996) [23]	RCC	Immunohist-ochemical (IHC)	39/45	87%	- increased expression at various levels in kidney cells of tumors with different histological and cytological properties; - c-Met may be involved in the formation and progression of renal cancer cells;
Pisters et al. (1997) [31]	Papillary cell	IHC	3/3	68%	- high expression of c-Met in renal cell carcinomas; - correlation between the higher level of c-Met and higher nuclear grade renal cancers;
	Mixed clear and granular cell		24/37		
	Rhabdoid/sarcomatoid		1/1		
	Oncocytoma	IHC	8/8	100%	
Sweeney et al. (2002) [45]	Papillary renal carcinoma	IHC	40/50	80%	20% of papillary cancers did not express c-Met—another possible mechanism responsible for tumorigenesis; - correlation of the growing stage of cancer with c-Met expression; - a tendency for higher overall survival in patients with c-Met -negative tumors;
Jong Sun Choi et al. (2006) [33]	Conventional renal carcinoma	IHC	43/96	45%	- significant relationship between c-Met expression and high nuclear level, as well as several clinical–pathological parameters, which indicates tumor invasion or aggressiveness; - the ability to distinguish between RCC subtypes by diffuse and strong immunoexpression of c-Met in RCC and collecting duct carcinomas as compared to subtypes with tubulo-papillary growth;
	Papillary renal carcinoma		18/20	90%	
	Chromophobe renal carcinoma		2/24	8%	
	Collecting duct carcinoma		5/5	100%	
	Urothelial carcinoma of renal pelvis		23/25	92%	
	Oncocytoma		0/12	0%	
Miyata et al. (2006) [24]	RCC	IHC	73/114	64%	- detection of pY1349 c-Met expression is an excellent predictor for the prognosis of patients with sporadic conventional RCC because it is positively associated with tumor grade, stage and size as well as cancer cell proliferation in patients;
Gontero et al. (2008) [46]	Papillary renal carcinoma	IHC	13/46	29%	- positive c-Met expression was significantly more common in papillary RCC (PRCC) type 2 (*p* < 0.001), however lack of correlation with multifocal kidney disease (*p* = 0.86) and occurrence of metachronous tumors (*p* = 0.93); - c-Met expression had no prognostic value;
Mukai et al. (2015) [47]	RCC	IHC	8/17	47%	- high c-Met and matriptase expression was found in RCC cells that had metastasized to bone and was accompanied by matriptase expression in osteoclasts, which indicates a significant role for these molecules in bone metastasis;
Macher-Goeppinger et al. (2017) [35]	ccRCC	IHC and CISH analyses	476/572	83%	- high expression of c-Met and *MET* copy number gains was associated with clinical and pathological features in the primary tumor, an aggressive phenotype and an unfavorable patient outcome; - c-Met expression and *MET* copy number should be used in relapses or metastases to target anti c-Met therapy in patients with ccRCC;
